# Recurrent Instability Rate and Subjective Knee Function following Accelerated Rehabilitation after ACL Reconstruction in Comparison to a Conservative Rehabilitation Protocol

**DOI:** 10.3390/jcm12144567

**Published:** 2023-07-09

**Authors:** Adrian Deichsel, Simon Oeckenpöhler, Michael J. Raschke, Ole Grunenberg, Christian Peez, Thorben Briese, Elmar Herbst, Christoph Kittl, Johannes Glasbrenner

**Affiliations:** Department of Trauma, Hand and Reconstructive Surgery, University Hospital Muenster, Albert-Schweitzer-Campus 1, Building W1, 48149 Münster, Germany; adrian.deichsel@ukmuenster.de (A.D.); michael.raschke@ukmuenster.de (M.J.R.);

**Keywords:** anterior cruciate ligament reconstruction, ACLR, rehabilitation, brace, weight-bearing

## Abstract

Introduction: The Purpose of the present study was to assess the outcome of anterior cruciate ligament reconstruction (ACLR) with an accelerated rehabilitation protocol and to compare it to a conservative rehabilitation protocol. It was hypothesized that an accelerated rehabilitation protocol, including brace-free early weight bearing, would result in a higher rate of recurrent instability and revision surgery compared to a conservative rehabilitation protocol. Methods: From 2016 to 2017, two different rehabilitation protocols for isolated ACLR were used at a high-volume knee surgery center. A total of 65 consecutive patients with isolated hamstring ACLR, of whom *n* = 33 had been treated with an accelerated (AccRehab) and *n* = 32 with a conservative rehabilitation protocol (ConRehab), were retrospectively included in the study. Patients were evaluated for recurrent instability, revision surgery, and other complications at a mean follow-up period of 64 ± 7.4 months. In addition, Tegner Activity Scale, Lysholm Score, and IKDC-subjective Score were evaluated. Statistical comparison between the two groups was performed utilizing Fisher’s exact test and Student’s t-test. Results: Mean age (29.3 vs. 26.6 years) and preoperative Tegner Score (6.4 vs. 5.9) were comparable between both groups. At 64 ± 7.4 months after ACLR, six cases of recurrent instability were reported in the AccRehab group (18%) in comparison to three cases (9%) in the ConRehab group (*p* = n.s.). There was no significant difference regarding revision surgery and further complications. Furthermore, no significant difference was found between both groups regarding Tegner (5.5 ± 1.9 vs. 5.5 ± 1.2), Lysholm (93.6 ± 6.3 vs. 89.3 ± 10.7), and IKDC score (89.7 ± 7.9 vs. 86.7 ± 12.1). Conclusion: No significant disadvantage of an accelerated rehabilitation protocol following ACLR was found in terms of recurrent instability rate, revision surgery, or patient-reported outcome. However, a trend towards a higher reinstability rate was found for an accelerated rehabilitation protocol. Future level one trials evaluating brace-free early weight bearing following ACLR are desirable.

## 1. Introduction

Despite advances in the surgical treatment of anterior cruciate ligament (ACL) injuries, recurrent instability following ACL reconstruction (ACLR) occurs in 5% to 20% of patients, with younger and physically active patients at an even higher risk of recurrent instability [1,2,3]. While the influence of surgical factors such as graft selection [4,5], tunnel positioning [6], and additional peripheral ligament procedures [7] have been extensively studied, there is little evidence regarding postoperative rehabilitation protocols and their influence on long-term outcomes of ACLR.

Different phases of rehabilitation following ACLR have been defined [8,9,10]. During the early postoperative period, pain-free, full range of motion (ROM) with safe walking is paramount, whereas late rehabilitation focuses on the restoration of full knee function and safe return to work, sport, and competition [11,12]. During the early postoperative period (up to 12 weeks), anti-inflammation and protection of the tendon graft during ligamentization must be balanced with a potential loss of range of motion and hypotrophy of the active stabilizers [10]. Common factors to consider during early postoperative rehabilitation are partial weight-bearing [13,14], the use of a extended or a hinged brace [15,16], and passive or active movement as well as graduated muscle training [17].

However, a variety of different rehabilitation protocols are currently used by knee surgeons following ACLR [13,18]. According to a recent systematic review, accelerated rehabilitation is defined as the early unrestricted range of motion and early brace-free full weight-bearing [17], whereas conservative rehabilitation protocols are characterized by restricted postoperative motion, the use of a brace, limited weight bearing, and delayed initiation of resistance exercises [19]. Nevertheless, there is no consensus on the optimal rehabilitation protocol for ACLR and there is limited evidence regarding the outcomes of accelerated rehabilitation protocols.

Therefore, the purpose of the present study was to compare the long-term outcome following isolated ACLR with a hamstring tendon autograft, in dependence of the rehabilitation protocol. It was hypothesized that an accelerated rehabilitation protocol including brace-free early weight bearing would result in a higher rate of recurrent instability and revision surgery compared to a conservative rehabilitation protocol.

## 2. Methods

### 2.1. Inclusion of Patients

Promising results of accelerated rehabilitation led to the implementation of an accelerated rehabilitation protocol for ACLR. During 2016 and 2017, two different rehabilitation protocols were used at the study center for patients undergoing isolated ACLR with an ipsilateral four-strand hamstring tendon graft. In the present study, consecutive patients between 18 and 60 years of age with isolated ACLR with hamstring tendon autograft in 2016 and 2017 were identified from the study center’s clinical database and included in the study if full consent for the follow-up interview was obtained. Patients with concomitant lesions such as meniscal tears and collateral ligament injuries that would alter the postoperative rehabilitation protocol were excluded. Similarly, those patients with previous knee injuries in both the affected and contralateral knee were excluded. Furthermore, meniscal repair or additional procedures such as lateral extra-articular tenodesis resulted in exclusion from the study.

### 2.2. Surgical Technique

All surgeries had been performed under general anesthesia, with single-shot perioperative antibiotic prophylaxis, by a board-certified surgeon with expertise in ACLR (at least >50 ACLR per year). Anatomic single-bundle ACLR was performed using a four-strand hamstring tendon (semitendinosus) autograft with a femoral fixed-loop suture button fixation and tibial hybrid fixation (interference screw and cortical suture button) [20]. Debridement or partial resection of small meniscal lesions was performed concomitantly with ACLR. There were no intraoperative complications in either group. At the end of surgery an intraarticular drainage was left for 24 h, and the knee joint was immobilized in a rigid brace at 0° for five days.

### 2.3. Accelerated Rehabilitation Protocol

A co-contraction routine of the quadriceps and hamstrings was practiced from the first day after surgery. Five days after surgery, a brace-free rehabilitation protocol was initiated, including non-weight bearing exercises without limitation in range of motion, full weight bearing was allowed, and progressive training using closed-chain knee exercises was started. Starting four weeks postoperatively, proprioceptive exercises were performed, including dynamic stability training. Running on a level surface was allowed after six weeks, and participation in pivoting sports was allowed after at least eight months and upon successful completion of a return-to-sports test.

### 2.4. Conservative Rehabilitation Protocol

After four days of knee immobilization in full extension, patients were instructed to wear a hinged hard frame brace for a total of six weeks with a range of motion limited to 90° of flexion and full extension. After two weeks of partial weight bearing with 20 kg, patients were instructed to slowly increase weight bearing with the goal of achieving full weight bearing at six weeks postoperatively. Progressive resistance training using closed-chain kinetic exercises, followed by proprioceptive exercises, was started at six weeks. Straight-line running was allowed at twelve weeks. Pivoting and competitive sports were allowed after at least eight months and upon successful completion of a return-to-sports test. The differences between the two rehabilitation protocols are described in Table 1.

### 2.5. Follow-Up

Informed consent to participate in the study was obtained from each patient, and epidemiologic data, as well as surgical measures and incidents during inpatient care, were recorded from the database. Patients were interviewed at a minimum follow-up of five years after surgery. Revision surgery, episodes of giving ways, and subjective instability were recorded. Patients were asked about the occurrence of other complications like joint infection, limited range of motion, or thrombotic events. If a complication occurred, the time after primary ACLR at which the complication occurred was recorded. In addition, the Tegner [21] activity scale, the International Knee Documentation Committee (IKDC) subjective knee form (IKDC-skf) [22], and the Lysholm score [23] were recorded. The visual analog scale from 0 to 10 points was used to determine the patient’s satisfaction with the outcome of the surgical procedure (10 indicating complete satisfaction).

### 2.6. Sample Size Calculation and Statistical Analysis

An a priori power analysis was performed using G*Power (version 3.1, University of Düsseldorf, Düsseldorf, Germany) [24]. A total sample size of *n* = 164 was calculated to obtain a power of 80% at the *p* = 0.05 significance level to detect a large effect size when utilizing Fisher’s exact test, based on potential differences in the rate of recurrent instability, as supported by the literature [25,26].

Statistical analysis was performed using PRISM (version 8, GraphPad Software, San Diego, CA, USA). Results are presented as means and standard deviations (SD). Criteria for normal distribution of numerical data were checked using histograms and the Shapiro–Wilk test. Normally distributed data were compared using Student’s *t*-test, while non-normally distributed data were compared using the Mann–Whitney test. Frequencies were compared by using Fisher’s exact test. A *p*-value < 0.05 was considered significant.

## 3. Results

### 3.1. Epidemiological Characteristics

A total of 65 patients with isolated ACLR with hamstring tendon autograft in 2016 to 2017, of whom *n* = 33 had been treated with an accelerated (AccRehab), and *n* = 32 with a conservative rehabilitation protocol (ConRehab), agreed to participate in the follow-up examination. No significant difference regarding epidemiologic data was found between the two groups at the time of the initial operation (see Table 2).

### 3.2. Recurrent Instability and Revision Surgeries

At a mean follow-up period of 64 ± 7.4 months, recurrent instability following ACLR had occurred in 3/32 cases (9%) in the ConRehab group and in 6/33 cases (18%) in the AccRehab group (*p* = n.s.). New trauma during motion was described in all three cases in the ConRehab group and in five of six cases in the AccRehab group. Recurrent instability occurred at 16, 22, and 47 months postoperatively in the ConRehab group and at 9, 10, 12, 12, 20, and 26 months postoperatively in the AccRehab group. Treatment of the recurrent instability was by revision ACLR in two of three cases in the ConRehab group and five of six cases in the AccRehab group.

Arthroscopic arthrolysis for a limited range of motion was performed in four patients in the ConRehab group, whereas no case of arthrofibrosis was observed in the accelerated group (*p* = n.s.). Other complications included deep vein thrombosis treated with pharmacologic anticoagulation in one patient in the ConRehab group, in comparison to one contralateral ACL injury and one secondary medial meniscus lesion in the AccRehab group.

In total, the number of revision surgeries necessary was in seven cases in the ConRehab group and five cases in the AccRehab group.

### 3.3. Patient Reported Outcome Measures (PROMs)

At 64 ± 7.4 months postoperatively, no significant differences were found between the AccRehab and the ConRehab group regarding Tegner (5.5 ± 1.9 vs. 5.5 ± 1.2), Lysholm (93.6 ± 6.3 vs. 89.3 ± 10.7) and IKDC subjective scores (89.7 ± 7.9 vs. 86.7 ± 12.1). Furthermore, there was no significant difference in patient satisfaction (8.5 ± 1.2 vs. 8.1 ± 2.5). The functional outcome is shown in Table 3.

## 4. Discussion

The most important finding of the present study was that an accelerated rehabilitation protocol with brace-free, early weight bearing after hamstring autograft ACLR, did not result in a significant difference in terms of recurrent instability, revision surgery, and patient-reported outcome compared to a conservative rehabilitation protocol. However, recurrent instability occurred in 18% (6/33) of the AccRehab group in comparison to 9% (3/32) in the ConRehab group, demanding a critical appraisal.

The aim of accelerated rehabilitation protocols for ACLR is to achieve early independent daily living with earlier return to work and physical activity without increasing the risk of insufficient graft healing and recurrent instability. However, on the one hand, there is a paucity of studies comparing current rehabilitation protocols for ACLR; on the other hand, there is a variety of accelerated rehabilitation protocols with heterogeneity in the available scientific data [27,28].

The failure rate of isolated ACLR is typically reported to range from 5 to 10% [26,29]. A systematic review summarizing the available data reported the overall failure rate of ACL reconstructions to be 11.9% [30]. However, multiple studies found that younger age and participation in high-risk pivoting sports (e.g., soccer) might increase the risk of recurrent instability [2]. A systematic review and meta-analysis aggregating 19 studies found the secondary ACL injury rate in patients under 25 years of age to be 21% [1]. If a patient returned to his/her pre-injury sporting level, the risk for secondary ACL injury increased even more, to 23%. A cohort study investigating 750 patients found that age under 20 years, as well as a successful return to sports, was associated with increased re-injury rates, up to 29%, at a mean follow-up of 4.8 years [2]. Furthermore, young age was associated with an increased risk of concomitant injuries like meniscal lesions with a negative impact on long-term outcomes [3]. Accordingly, in the present study, young patient age (mean 29.3 and 26.6 years) and high physical demand (mean Tegner score 6.4 and 5.9) might have contributed to the rate of recurrent instability in the AccRehab (18%) and the ConRehab group (9%). Although no statistically significant difference was found between the two groups, recurrent instability occurred within the first 12 postoperative months in 4 of 6 cases of the AccRehab group, whereas the three cases of recurrent instability in the ConRehab group occurred at 16, 22 and 47 months postoperatively. Therefore, a tendency towards higher risk of recurrent instability due to accelerated rehabilitation might be suspected. However, sample size in both groups was lower than calculated by the power analysis for recurrent instability (*n* = 164 required, *n* = 65 achieved). Therfore, the trend towards increased recurrent instability in the AccRehab groupd might have resulted erroneously non-significant in the present study. However, future trials with larger sample sizes on this issue are desirable.

A hard frame brace is used in the majority of rehabilitation protocols following ACLR to reduce strain on the reconstructed ligament [31]. However, the influence of postoperative bracing remains unclear due to a lack of comparative studies [32]. In a recent randomized controlled trial (RCT) of 114 patients, brace-free rehabilitation after hamstring ACLR was found to result in equivalent postoperative PROM scores at the one-year follow-up compared with a rehabilitation protocol that included a brace for 6 weeks [16]. A retrospective study of 969 patients found no significant benefits in terms of complications for patients who wore a brace postoperatively [33]. A recent meta-analysis concluded that brace wear after isolated ACLR does not significantly influence postoperative outcomes toward improvement [34]. However, in a recent survey of American surgeons, 82.6% of respondents reported using a brace postoperatively to aid in rehabilitation. The reason for this persistent trend, however, could not be further elucidated.

A limited range of motion has been reported in 4 to 38% of patients after knee ligament surgery [35]. It has previously been shown that prolonged immobilization or limited weight bearing may lead to increased rates of limited ranges of motion [36]. In the present study, full weight bearing was initiated after 5 days in the AccRehab group, whereas partial weight bearing with 20 kg body weight was recommended for two weeks in the ConRehab group, with subsequent step-wise increased weight bearing. A trend towards limited ranges of motion in the ConRehab group was noted, with four patients needing arthroscopic arthrolysis during the follow-up period. In contrast, no case of arthrofibrosis was found in the AccRehab group. The difference in the incidence of arthrofibrosis between the groups, however, was not statistically significant. These findings are consistent with a prospective study of more than 200 patients who underwent either progressive rehabilitation with full weight bearing after four weeks or a delayed rehabilitation with full weight bearing after eight weeks, which found no significant differences in the occurrence of reduced range of motion between the groups [37]. Some studies have hypothesized that early weight bearing and early inclusion of progressive resistance training may lead to a faster recovery of knee function compared with conservative rehabilitation. However, a study comparing immediate full weight bearing with two weeks of non-weight bearing found no significant differences in the range of motion and anteroposterior knee stability [14]. A prospective study comparing the complete unloading of the operated extremity for either one or two weeks found no significant differences in PROMs, anteroposterior knee laxity, or incidence of tunnel widening at 12 months [38]. A systematic review with meta-analysis comparing accelerated and delayed weight-bearing protocols found a significant advantage of early weight bearing regarding the IKDC (mean 7.3 points) at the 12- to 24-month follow-up, at the cost of a minimally increased anteroposterior laxity (mean 0.3 mm) [39]. Based on this evidence, and compared with the results of the current study, limited weight-bearing does not appear to have a beneficial effect in isolated ACLR, but might increase the risk for impaired postoperative range of motion.

PROMS after ACLR with an accelerated (no brace, immediate weight bearing as tolerated, free ROM) vs. non-accelerated rehabilitation protocol (immobilizer brace for four weeks, two weeks of limited weight bearing) was evaluated in a recent randomized controlled trial (RCT). The authors found no significant difference in subjective IKDC score, range of motion, and peak quadriceps isometric strength at 12 and 24 weeks [28]. In another RCT, the comparison of an accelerated and a conservative rehabilitation protocol after hamstring ACLR showed a significant advantage of the accelerated protocol regarding the Lysholm score at three and six months and the Knee Injury and Osteoarthritis Outcome score (KOOS) at six months, without a significant increase in anteroposterior knee laxity [40]. Similar findings were reported by the authors of an RCT comparing accelerated and conservative rehabilitation after ACLR with a bone–patellar-tendon–bone (BPTB) graft [27]. A recent systematic review of studies comparing accelerated and non-accelerated rehabilitation protocols after hamstring ACLR was reported in seven studies, of which four were RCTs [19]. Although a small advantage was found for the non-accelerated group in anteroposterior knee stability (MD 0.59 mm; 95% CI 0.12–1.07 mm) and tunnel widening (MD 0.48 mm; 95% CI 0.00–0.96 mm), the differences between the two groups were not clinically relevant. Significantly better PROMS were reported in the accelerated group at 3 and 6 months postoperatively, but again without surpassing the minimal clinically important difference (MCID). These findings are consistent with the results of the present study, in which no significant differences in Tegner, Lysholm and IKDC-subjective scores were found between the two groups.

The results of the present study are of clinical relevance, considering that ACL reconstruction is the most frequent ligament surgery in Western countries, leading to socioeconomically favorable results, especially in young, physically active patients [41,42,43]. Optimizing rehabilitation after ACLR might improve the positive effect of ACL reconstruction by allowing earlier unrestricted motion of the operated joint, possibly allowing for accelerated return to social activities, work, and sports, while simultaneously protecting from re-injury of the operated joint [13,44,45].

However, besides the rehabilitation protocol, several factors should be considered, possibly influencing the postoperative outcome and reinstability rate following ACLR. Body mass index (BMI) was shown to be a major influence factor, with obese patients resulting in inferior clinical outcomes after ACL reconstruction in comparison to non-obese patients [46]. Furthermore, the chosen tendon graft might influence the reinstability rate, especially in the young population. In a cohort study of over 800 patients aged 17 to 22 participating in pivoting sports, ACL reconstruction utilizing four-strand hamstring tendons, as in the present study, was associated with increased failure rates (odds ratio 2.1), in comparison to bone-patellar tendon-bone (BTB) autografts [47]. In these patients, quadriceps or BTB autografts might be a preferable option [48]. Additional stability of the operated knee might be achieved by adding a lateral extra-articular tenodesis to the ACL-reconstructed knee to reduce anterolateral rotatory instability, as assessed with the pivot-shift test [49]. A recent multicenter RCT investigating the effect of lateral extra-articular tenodesis, in addition to hamstring autograft ACL reconstruction, in young patients with a high risk for re-injury, showed a risk reduction of 67% in ACL graft failure when a tenodesis was applied [7]. Lastly, the posterior tibial slope, defined as the inclination of the tibial plateau in the sagittal plane, was recently shown to be a major contributor to ACL injury and re-injury. A study investigating 519 patients with ACL graft insufficiency found a posterior tibial slope of over 12 degrees to massively increase the risk for repeated graft insufficiency (odds ratio 11.6) [50].

Several limitations should be considered when transferring the results of the present study into clinical practice. First and foremost, the inclusion of patients was performed retrospectively, which makes the inclusion process prone to selection bias. However, the comparability of baseline epidemiological parameters and selection from a single center in the same time frame minimizes selection bias. Unfortunately, the results of this study are underpowered regarding the primary outcome (reinstability rate) of the study. Therefore, it is possible that a true difference between the groups was not significant due to the small sample size. Therefore, it is desirable that further clinical studies investigate accelerated rehabilitation protocols on prospective clinical studies with larger sample sizes.

It has previously been hypothesized that telephone assessment of PROMs may influence the data obtained with a possible bias towards higher values [51]. However, a recent study comparing telephone and on-site assessments of the Lysholm score during follow-up found no significant difference between groups [52]. Another limitation of the present study is that outcomes were assessed only at the long-term follow-up. Possible differences in knee function in the short- and mid-term could not be considered. Furthermore, exact adherence to the prescribed rehabilitation protocol could not be controlled due to the retrospective nature of the study.

## 5. Conclusions

No significant disadvantage of an accelerated rehabilitation protocol following ACLR was found in terms of recurrent instability rate, revision surgery, or patient-reported outcome in the present study. However, a tendency towards a higher rate of recurrent instability when utilizing an accelerated rehabilitation protocol indicates that level one trials evaluating brace-free early weight bearing following ACLR are desirable.

## Figures and Tables

**Table 1:** Comparison of the accelerated and conservative rehabilitation protocol.

	AccRehab	ConRehab
Immobilization	4 days	4 days
Partial weight bearing	4 days	2 weeks
Hard frame brace	-	6 weeks (0–0–90°)
Resistance exercises	after 2 weeks	after 6 weeks
Running	after 6 weeks	after 3 months
Competitive sports	after 8 months	after 8 months

AccRehab, accelerated rehabilitation protocol; ConRehab, conservative rehabilitation protocol.

**Table 2:** Preoperative epidemiological data of both study groups.

	AccRehab (*n* = 33)	ConRehab (*n* = 32)	*p*-Value
Age	29.3 (SD 11.7)	26.6 (SD 11.7)	n.s.
Gender (female/male)	8/25	18/14	n.s.
Affected side (left/right)	15/18	12/20	n.s.
BMI (kg/m^2^)	24.1 (SD 2.6)	23.6 (SD 3.9)	n.s.
Smokers (yes/no)	3/30	1/31	n.s.
Tegner Score at Injury	6.4 (SD 1.3)	5.9 (SD 1.6)	n.s.

AccRehab, accelerated rehabilitation protocol; ConRehab, conservative rehabilitation protocol.

**Table 3:** Comparison of patient-reported outcome measures between both study groups.

	AccRehab	ConRehab	*p*-Value
Tegner Activity Scale (0–10)	5.5 ± 1.9	5.5 ± 1.2	n.s.
Lysholm Score (0–100)	93.6 ± 6.3	89.3 ± 10.7	n.s.
IKDC subjective Score (0–100)	89.7 ± 7.9	86.7 ± 12.1	n.s.
VAS Patient Satisfaction	8.5 ± 1.2	8.1 ± 2.5	n.s.

Values are presented as mean and standard deviation; AccRehab, accelerated rehabilitation protocol; ConRehab, conservative rehabilitation protocol.

## Data Availability

The data are not publicly available due to privacy of the included patients.
